# Full Efficiency Recovery in Hole-Transporting Layer-Free Perovskite Solar Cells With Free-Standing Dry-Carbon Top-Contacts

**DOI:** 10.3389/fchem.2020.00200

**Published:** 2020-04-17

**Authors:** Salvatore Valastro, Emanuele Smecca, Salvatore Sanzaro, Ioannis Deretzis, Antonino La Magna, Youhei Numata, Ajay Kumar Jena, Tsutomu Miyasaka, Antonio Gagliano, Alessandra Alberti

**Affiliations:** ^1^CNR-IMM, Catania, Italy; ^2^Department of Electrical, Electronic and Computer Engineering, University of Catania, Catania, Italy; ^3^Graduate School of Engineering and Faculty of Medical Engineering, Toin University of Yokohama, Yokohama, Japan

**Keywords:** solvent-free, durability, stability, nitrogen, MAPbI_3_, self-curing, healing

## Abstract

Carbon-based top electrodes for hole-transporting-layer-free perovskite solar cells (PSCs) were made by hot press (HP) transfer of a free-standing carbon-aluminum foil at 100°C and at a pressure of 0.1 MPa on a methylammonium lead iodide (MAPbI_3_) layer. Under these conditions, the perovskite surface was preserved from interaction with the solvent. Over a timescale of 90 days, HP-PSCs were systematically compared to reference cells with carbon-based top electrodes deposited by doctor blading (DB). We found that all the photovoltaic parameters recorded in HP-PSCs during time under ambient conditions settled on values systematically higher than those measured in the reference DB-PSCs, with efficiency stabilized at around 6% within the first few measurements. On the other hand, in DB-PSCs, a long-lasting (~14 days) degrading transient of the performances was observed, with a loss of efficiency from an initial ~8% to ~3%. Moreover, in HP-PSCs, a systematic day-by-day recovery of the efficiency after operation was observed (Δ~2%) by leaving the cell under open circuit, a nitrogen environment, and dark conditions. Noteworthily, a full recovery of all the parameters was observed at the end of the experiment, while DB-PSCs showed only a partial recovery under the same conditions. Hence, the complete release of solvent from the carbon contact, before an interface is established with the perovskite layer, offers a definite advantage through the long period of operation in preventing irreversible degradation. Our findings indeed highlight the crucial role of the interfaces and their feasible preservation under nitrogen atmosphere.

## Introduction

Perovskite solar cells (PSCs) based on organometal halide perovskite materials have shown a rapid development since their birth in 2009, when Miyasaka et al. (Kojima et al., 2009) applied hybrid perovskites in a photo-to-energy conversion device that achieved an efficiency of 3.8%. Recently, a maximum photo-conversion efficiency (PCE) of 25.2% ^1^ has been added in the NREL chart (07/2019).

The commercialization of PSCs is primarily hindered by the low structural stability of the active material (Alberti et al., 2015, 2019a; Ciccioli and Latini, 2018; Deretzis et al., 2018; Miyasaka, 2018; Sanzaro et al., 2019) and the limited durability of the devices (Jena et al., 2019). Different from what occurs on more consolidated technologies (e.g., those Si-based), perovskite material preservation along the operation time represents a huge concern from many different perspectives. As recently reported in the literature (Jena et al., 2019), moisture action (Mosconi et al., 2015; Müller et al., 2015; Wang et al., 2017), intrinsic lattice defects (Tejas et al., 2017), light soaking, ionic migration (Aristidou et al., 2017), and interface coupling of materials (Alberti et al., 2019c) can have a deleterious impact on the electrical and structural behavior of the perovskite layer. In addition, the atomic interaction at the interface between perovskite and the hole-transporting layer (HTL) has played a crucial role in the worsening of device performances. This is primarily due to progressively induced physical/chemical changes and contaminant diffusion, further promoted by the specific working conditions (e.g., temperature increase under operation), while degradation of the core of the perovskite layer is expected to have a minor effect (Jena et al., 2019).

In recent years, HTL-free carbon-based PSCs (Cai et al., 2017) have been developed as an alternative to the more expensive and less stable (Jena et al., 2018) Spiro-OMeTAD-based perovskite solar cells. Their use is currently limited by the insufficient hole selectivity and by the difficult coupling between the carbon electrode and the perovskite, resulting in unsatisfying PCEs. On the other hand, the perspective of gaining durability by stabilizing the carbon/perovskite interface (Chen and Yang, 2019), and preserving the perovskite from the environmental humidity is raising the expectation for this technological solution. This is mainly due to the structural stability and the hydrophobic character of the carbon-based material, making it a promising back-contact (it is opaque) for commercialization of PSCs. The most-applied carbon materials are carbon pastes that are usually composed by carbon powder (graphite and carbon black), curing resin, and solvent. Solvent release from the material and its interaction with the perovskite layer represent critical issues that need to be properly addressed (Wei et al., 2015; Zhang et al., 2018).

Several carbon electrode fabrication methods can be used. Among the others, doctor blading and hot press transfer (Wei et al., 2015; Zhang et al., 2018) represent two up-scalable, viable approaches for carbon electrode deposition. Hot press transfer of C-based foils was recently applied on Spiro-OMeTAD as an alternative to metal-based back electrodes. As a drawback, an effort is needed to preserve the HTL from damage during heating (Zhang et al., 2018). Thereby, press procedures without heating were applied in combination with Spiro-OMeTAD (RT, *P* = 0.70 MPa). In a different approach and to reduce the device cost, carbon contacts were made directly on the perovskite layer by hot pressing (85°C, *P* = 0.15–0.40 MPa) (Wei et al., 2015), with reduced selectivity of the contact to hole extraction. In this respect, the balance of costs and benefits in quality and durability of the created Carbon/Perovksite interface is currently not fully unveiled. Moreover, a systematic comparison between the most-used doctor blading and the hot press transfer procedures is currently lacking in the literature, especially focusing on the durability of the carrier extraction action. Gaining further insights on how the carbon electrodes interact with the perovskite layer would open the field to a more effective optimization of the interfaces with impact on the device reliability over time.

Herein, we compare the durability over months of HTL-free carbon-based PSCs, realized on MAPbI_3_ layers with two up-scalable top-contact fabrication methods—namely, doctor-blading and hot press transfer—by analyzing the evolution of the electrical parameters in devices. The hot press transfer is done at 100°C and at a pressure of 0.1 MPa—a compromise with respect to what is already explored in the literature (Wei et al., 2015; Zhang et al., 2018). This systematic investigation has highlighted a viable approach to avoid the deleterious action over time of the solvent present in commercially available C-based pastes. It is also demonstrated that a full recovery of the device performances is even feasible under specific conditions.

## Results and Discussion

**Figure 1:**
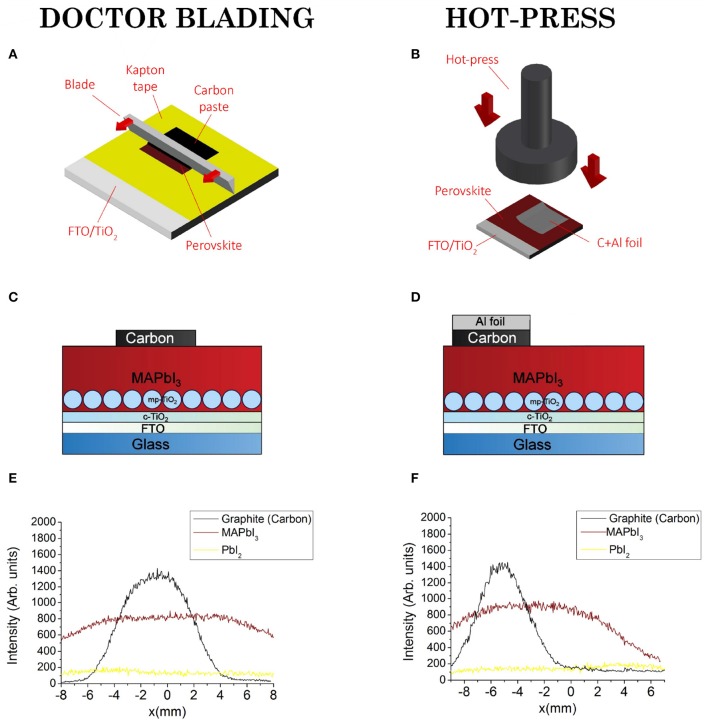
Doctor blading and hot press transfer methods for carbon electrode fabrication and compositional line-profiles. **(A)** Schematic illustrating doctor blading and **(B)** hot press methods. **(C,D)** Two devices architectures. **(E,F)** Line profiles taken by XRD through the contact regions.

The two methods used to fabricate carbon electrodes by doctor blading (DB) and hot pressing (HP) on twin MAPbI_3_/TiO_2_/FTO samples, and the related device structures, are shown in Figures 1A–D, respectively. Composition inside and outside the contact regions was unfolded by X-ray diffraction that probed the layered structure of the device architecture. During the analysis, the X-ray beam was scanned through the contact at fixed Bragg-angles of MAPbI_3_ (main peak at 2θ = 14.1°), PbI_2_ (main peak at 2θ = 12.7°) or graphite (main peak at 2θ = 26.55°). On the basis of this spatial-resolved investigation, the line profiles of MAPbI_3_, PbI_2_, and graphite (the main component in the commercial paste) are traced as shown in Figures 1E,F. In both samples, the PbI_2_ amount inside and outside the contact area is almost negligible meaning that no macroscopic degradation of the perovskite layer occurred during contact formation.

**Figure 2:**
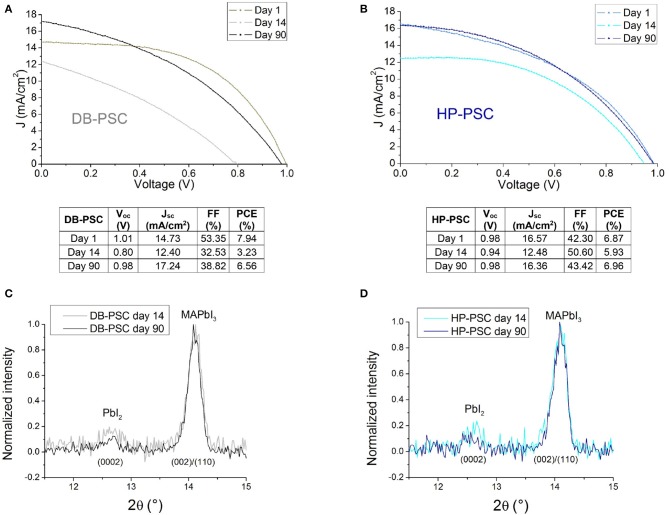
**(A,B)** J-V curves and photovoltaic parameters of representative DB-PSC and HP-PSC, respectively at the beginning (day 1) and at the end (day 90) of the cycle. Data related to day 14, taken as intermediate cases, are also shown to represent the photovoltaic response during recursive operation. **(C,D)** XRD patterns acquired at day 14 and day 90. Material recovery is represented by the relative reduction (normalised to the MAPbI_3_ peak maximum) of the PbI_2_ amount after prolonged storage in nitrogen, open circuit and dark conditions. The diffraction patterns are labelled with the related Miller indexes of the exagonal (PbI_2_) and tetragonal (MAPbI_3_) phases.

Figures 2A,B show the electrical response of as prepared devices (day 1) under 1 sun illumination. In particular, the DB-PSC shows better performances than the HP-PSC. We firstly argue a minor role of the Al foil used in the HP-PSCs (to be noticed the slope at the V_oc_). The best cell works at an efficiency of 7.94% with short-circuit photocurrent density (J_sc_) = 14.73 mA/cm^2^, open-circuit voltage (V_oc_) = 1.01 V, and fill factor (FF) = 53.35%. These values closely match the data reported in the literature for similar device architectures and commercial C-based pastes, as summarized in Cai et al. (2017). On the other hand, the starting efficiency of the HP-PSC is 1% less than the reference DB-PSC (6.87 vs. 7.94%). The discrepancy is verified over the statistics of four to five devices, being indeed not ascribable to a perovskite layer variability. This finding provides the first indication of a better initial contact between the DB-carbon electrode and the perovskite layer immediately after its production. It is indeed likely that, via DB, a chemical-assisted coupling of the C-electrode with the perovskite is nicely promoted by the solvent, as opposed to the case of hot pressing the C+Al-foil wherein the film is induced to a structural coupling by heat and pressure from the C-side. In countertrend, the short circuit current value is higher in HP-PSCs, and this likely helps the performance improvement over time discussed hereafter.

In order to evaluate how stable the device performances are, DB-PSCs and HP-PSCs were measured at regular intervals over 1 month, under 1 sun illumination, and in environmental atmosphere without any encapsulation. After each measurement, the devices were stored in nitrogen atmosphere under open-circuit and dark conditions.

The J-V curves at the beginning and end of operation, together with the curves taken at day 14, testify that a recovery of the performances can be achieved by both DB-PSCs and HP-PSCs (Figures 2A,B). It is noteworthy that in HP-PSCs the recovery allows getting back the initial J_sc_, V_oc_, and FF values; it is instead partial in DB-PSCs. The structural analysis in Figures 2C,D demonstrates that the recovery of the photovoltaic parameters corresponds to a slight reduction of the PbI_2_/MAPbI_3_ intensity ratio of the related diffraction peaks with respect to what measured in the same samples at day 14 (lower performances). This finding can be equally interpreted as an improvement of the perovskite lattice quality during time or as a reduction of the PbI_2_ amount (by a factor 0.6 from day 14 to day 90 in the HP-PSC) that would imply the overall mass (including the organic cations) preserved during the cycle of operation. The measured lattice improvement cannot be fully ascribed to compositional disuniformities along the sampled area, since this would produce smaller normalized variations in the PbI_2_/MAPbI_3_ peak intensity ratio (~0.1–0.2).

In conjunction with a recovery of the active material structure that was observed in both samples, the uncomplete restoring of the photovoltaic parameters in the DB-PSC with respect to what occurs in the HP-PSCs at the end of the cycle (day 90) addresses that the C/perovskite interface has been irreversibly modified.

**Figure 3:**
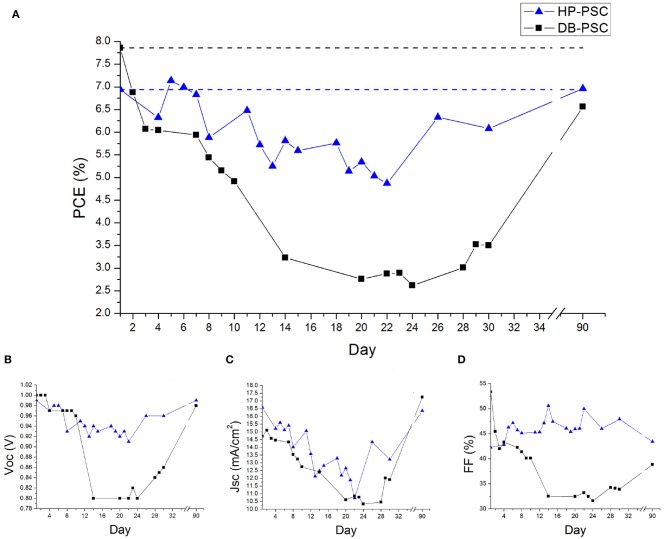
Temporal trend of the PCE values for representative DB-PSC and HP-PSC **(A)**. In both cases a recovery of the starting performances can be noticed, which is fully achieved for HP-PSC **(B–D)**.

Figure 3A shows the temporal trend of the PCE values collected in representative DB-PSC and HP-PSC. We notice that the efficiency of the DB-PSC has a long-lasting transient behavior consisting of a severe reduction until day 14. This suggests the occurrence of further structural changes and/or interfacial re-arrangements in proximity of the interface between the carbon contact and the perovskite layer, which was initiated with the intermediation of the solvent of the carbon paste at the curing temperature (120°C used here; similar results at day 1 using 100°C). On the other hand, a pronounced deterioration of the perovskite bulk is not expected at the used curing temperature on the basis of our previous investigation (Smecca et al., 2016). A similar transient behavior is observed for all the other photovoltaic parameters, namely V_oc_, J_sc_, and FF (Figures 3B–D). Although the nature of the solvent in the commercial paste is not specified, some hints can be drawn (Wei et al., 2015) on polarity- and steric hindrance-related effects (Sanzaro et al., 2017) in charge separation (e.g., by charge screening) and indeed in defect generation (e.g., iodide charged species) contributing to local structural changes. With limited mass loss, a recovery of the performances is feasible by a local structural rearrangement of the active material. At the structural side, the diffraction pattern in Figure 2C does not show any dramatic degradation of the perovskite layer under the contact after 14 days of operation. From day 14 to day 30, the device shows deterioration/recovery pathways and the PCE consequently fluctuates in the range 3–3.5%.

HP-PSCs show a different behavior. As a piece of first evidence, the device does not exhibit a marked transience in the PCE values after initial operation (Figure 3A). Moreover, after day 1, a path of deterioration and recovery is immediately initiated. In particular, the PCE values fluctuate in the range 5–6%, which is significantly above the one measured on the DB-PSC (fluctuating in the range 2.5–3.5%). Moreover, a medium-term quasi-full recovery of the device performance, not detected in DB-PSCs, is even achieved when the storage time is long enough (e.g., 5 days). This effect is shown in Figure 3A between days 22 and 26. It is finally noteworthy that storing the HP-PSC device without break for 60 days in nitrogen atmosphere under open-circuit and dark conditions (at day 90) allows a full recovery of the efficiency (6.96 %) and of all the other photovoltaic parameters (V_oc_, J_sc_, FF). This is clearly stated by Figures 3B–D.

On the opposite, the DB-PSC (Figure 3A) at day 90 has only a partial recovery of the efficiency (6.56 %) with respect to the value recorded at day 1 (7.94%). The specific behavior of V_oc_, J_sc_ and FF (Figures 3A–C) over time frames that a crucial role is played by the FF with its link to the carrier extraction and transport at the interfaces.

Although in a different scalebar, a deterioration/recovery behavior is feasible on both kinds of devices. This is a phenomenon likely linked to (1) migration of ions in the perovskite layer under illumination and electric field (partially reversible), (2) defects formation under operation and self-curing of the perovskite (Fakharuddin et al., 2017; Ceratti et al., 2018; Schulz et al., 2019) of the perovskite in absence of electric field and under dark condition, and (3) healing action by nitrogen (Mannino et al., 2017; Alberti et al., 2019b,d) on the perovskite and at the perovskite/carbon interface.

In this respect, the cross-correlation of data suggests that the release of solvent from the carbon paste before being in touch with the perovskite layer results in preserving the perovskite/carbon interface from irreversible changes. The partial reversibility of the DB-PSC performances can be instead linked to a permanent modification of the interface that is not fully recoverable during time. Above the full recovery of the HP-DSC, a further improvement of the cell efficiency is expected by engineering the contact (Chen and Yang, 2019) such to reduce the series resistance and increase contact selectivity. This is expected to primarily impact on the fill factor of the solar cells.

**Figure 4:**
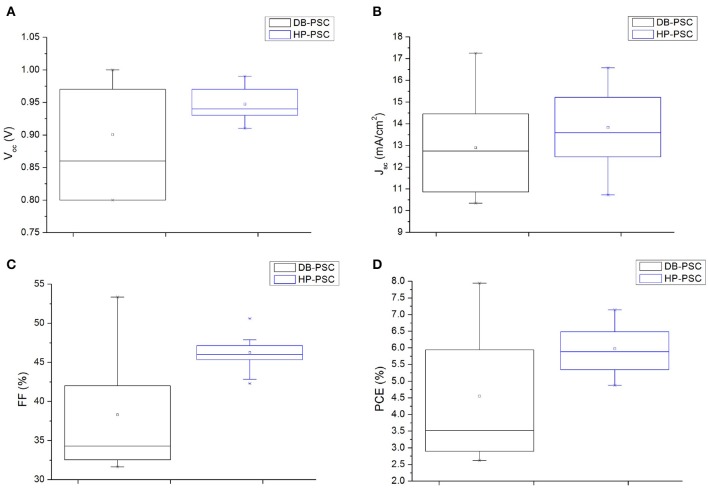
Box plots representing data distributions of Voc **(A)**, Jsc **(B)**, FF **(C)**, and PCE **(D)** for DB-PSCs and HP-PSCs.

The statistical distributions of the V_oc_, J_sc_, FF, and PCE values for the DB-PSCs and HP-PSCs over four to five devices taken along the full temporal scale of the experiment (90 days), including indeed all data along the degradation-recovery path, are summarized as box plots in Figure 4. We notice that all the values of the electrical parameters in DB-PSCs are more widely scattered than those of HP-PSCs, resulting in larger boxes. Moreover, the median values are higher in HP-PSCs. All those findings imply that HP-PSCs exhibit (1) a better reproducibility of the top-side electrical contact, (2) more reliable and improved performances, and (3) better long-term stability.

## Conclusions

Low temperature preparation of Carbon-based top-electrodes were done by hot press transfer method of a free-standing C+Al foil at 100°C and pressure of 0.1 MPa and integrated in PSCs architectures. HP-PSCs were systematically investigated during time by acquiring J-V curves in ambient conditions. After operation, the cells were left under open circuit voltage, nitrogen environment, and dark conditions. Limited deterioration and significant day-by-day recovery were observed along the entire timescale, which suggests the carbon/perovskite interface is sufficiently preserved from degradation during operation, as well as a capability for self- and nitrogen-assisted healing of the perovskite layer while not operating. This capability is definitely demonstrated by the full recovery of all the device parameters, primarily the efficiency, at the end of the experiment. On the opposite, the reference DB-PSC exhibited a monotonic descending trend of the performances during time that has drawn a transient behavior driven by degradation. The action of solvent during contact formation and its possible further action during time is indeed demonstrated to have deleterious impact on the cell performances in the long period. Solvent-based deposition methods for the C-contact indeed demand tailored procedures to mitigate the negative effects. We additionally argue that, above the full recovery of the HP-PSCs performances, a further increase of the efficiency would be feasible by improving the selectivity of the C-contact and the conductivity of the overall top-electrode.

## Experimental Section

### MAPbI_3_ Synthesis and Deposition

A 450 nm-thick MAPbI_3_ layer was deposited by solution processing on a 90 nm-thick meso-porous-TiO_2_ on compact-TiO_2_/FTO. Before preparation of perovskite film, a perovskite precursor solution and the substrates were warmed at 70°C. A 1.5 M solution of methylammonium iodide (MAI) and PbI_2_ (1: 1 in mol) in *N, N*-dimethylformamide (DMF) and dimethylsulfoxide (DMSO) (4:1 v/v) was poured onto the TiO_2_ mesoporous substrate and spin-coated (kept for 60 sec; 5,000 rpm for 30 s with chlorobenzene dripping). The as-prepared film was pot-roast vapor annealing (PR-VA)-treated at 100°C for 10 min on a hot plate. The resulting perovskite layer was compact and uniform. The size of the grains was in the range 400–800 nm.

### C-Based Top Electrode Deposition

To complete PSC architecture, a carbon layer was deposited following two methods. In the doctor blading method, a commercial carbon paste (Dyenamo DN-CP01) was coated onto the perovskite layer with a blade. A kapton tape frame is used to define the area (*A* = 0.25 cm^2^) and thickness (30 μm) of the top electrode. Then, the PSC with the wet carbon film was dried at 120°C for 15 min (according to the Dyenamo datasheet) in nitrogen atmosphere to remove the solvent and to promote the curing of the paste. Trials by using 100°C annealing produced similar performances in devices.

In the hot press transfer method, glass substrate was coated with the commercial carbon paste by doctor blading, forming a wet carbon film. Then it was soaked into ethanol for a solvent-exchange. After that, the carbon film was peeled off from the glass substrate and, differently from ref (Zhang et al., 2018) that leaves the contact free-standing, it was dried at RT on an aluminum foil. As a result, a C+Al free standing electrode (*A* = 0.25 cm^2^) was formed and directly hot pressed onto the perovskite layer under the pressure of 0.1 MPa at 100°C for 40 min.

### X-ray Diffraction

X-ray diffraction analyses were performed using a high angular resolution diffractometer made by Bruker AXS equipped with a Cu-kα source, soller slits, and a Goebel mirror. Line profiles were acquired at a fixed Bragg angle using a beam size of 1 cm × 1 mm that was moved through the contact area using a motorized sample stage. This allows drawing the region wherein the compound/species producing that specific Bragg peak is spatially located.

### Device Characterization

J-V characteristics were measured by a digital source meter (Keithley model 2401) under AM 1.5-simulated sunlight (100 mW/cm^2^) from Peccell PEC-L01. The solar cells devices were masked (shadow mask) to define the active area of 0.25 cm^2^ and then irradiated from the backside at the FTO/TiO_2_ side. Scan range was from −0.1 to 1.2 V for forward scan and from 1.2 to −0.1 V for reverse scan with a step of 0.01 V. The delay time was of 0.1 s.

## Data Availability Statement

The raw data supporting the conclusions of this article will be made available by the authors, without undue reservation, to any qualified researcher.

## Author Contributions

SV deposited the C-based contact, performed the electrical characterization of the devices, and wrote the paper draft. ES performed the X-ray characterization of the devices. SS prepared the free-standing C-foils. YN and AJ prepared the TiO_2_/perovskite bilayers. ID, AL, AG, and TM made supervision actions. AA conceived the experiment, coordinated the activities, and refined the paper.

## Conflict of Interest

The authors declare that the research was conducted in the absence of any commercial or financial relationships that could be construed as a potential conflict of interest.

## Footnotes

^1^https://www.nrel.gov/; viewed in 07-2019.
